# Structure and Transport Properties of the BiCuSeO-BiCuSO Solid Solution

**DOI:** 10.3390/ma8031043

**Published:** 2015-03-12

**Authors:** David Berardan, Jing Li, Emilie Amzallag, Sunanda Mitra, Jiehe Sui, Wei Cai, Nita Dragoe

**Affiliations:** 1 SP2M—ICMMO, Université Paris-Sud, Orsay F-91405, France; E-Mails: lijing35101@126.com (J.L.); emilie.amzallag@u-psud.fr (E.A.); sunanda.mitra@u-psud.fr (S.M.); nita.dragoe@u-psud.fr (N.D.); 2 School of Materials Science and Engineering, Harbin Institute of Technology, Harbin 150001, China; E-Mails: suijiehe@hit.edu.cn (J.S.); weicai@hit.edu.cn (W.C.)

**Keywords:** thermoelectric materials, layered chalcogenides, crystal structure, electronic band structure, thermal conductivity, electrical resistivity

## Abstract

In this paper, we report on the crystal structure and the electrical and thermal transport properties of the BiCuSe_1−x_S_x_O series. From the evolution of the structural parameters with the substitution rate, we can confidently conclude that a complete solid solution exists between the BiCuSeO and BiCuSO end members, without any miscibility gap. However, the decrease of the stability of the materials when increasing the sulfur fraction, with a simultaneous volatilization, makes it difficult to obtain S-rich samples in a single phase. The band gap of the materials linearly increases between 0.8 eV for BiCuSeO and 1.1 eV in BiCuSO, and the covalent character of the Cu-*Ch* (Ch = chalcogen element, namely S or Se here) bond slightly decreases when increasing the sulfur fraction. The thermal conductivity of the end members is nearly the same, but a significant decrease is observed for the samples belonging to the solid solution, which can be explained by point defect scattering due to atomic mass and radii fluctuations between Se and S. When increasing the sulfur fraction, the electrical resistivity of the samples strongly increases, which could be linked to an evolution of the energy of formation of copper vacancies, which act as acceptor dopants in these materials.

## 1. Introduction

In recent years, BiCuSeO-based oxychalcogenides have been suggested as promising thermoelectric materials for applications in thermal energy conversion in a 350 °C–650 °C temperature range [1,2]. These materials crystallize in a tetragonal structure with P4/nmm space group, the same ZrCuSiAs structure type as the 1111 iron-based superconductors [3,4], which consists of [Bi_2_O_2_] and [Cu_2_Se_2_] layers alternatively stacked along the *c*-axis of the unit cell [5,6,7]. It has been shown using electronic band structure calculations that the electrical properties are mostly driven by the [Cu_2_Se_2_] layer [8,9], which consists of slightly distorted CuSe_4_ tetrahedra linked by their edges. When unintentionally doped, they are moderate band gap semiconductors (E_g_~0.8 eV, in the case of BiCuSeO), and they exhibit only moderate electrical conductivity values (σ~10 S·cm^−1^ in the case of BiCuSeO at room temperature) with p-type conduction [8,9,10]. However, they can be very easily p-type doped by substituting Bi^3+^ with a 2+ (Ba^2+^, Ca^2+^, Pb^2+^ or Sr^2+^) [9,10,11,12,13,14,15,16] or a 1+ (K^+^ or Na^+^) [17,18] element, or by the controlled introduction of copper vacancies [19]. This doping leads to a simultaneous decrease of the electrical resistivity and the Seebeck coefficient [9], and the thermoelectric power factor, defined as PF = S^2^/ρ (with S as the Seebeck coefficient and ρ the electrical resistivity), reaches about 0.7 × 10^−3^ W·m^−1^·K^−2^ in optimally doped samples [11]. This value is much lower than the ones observed in state-of-the-art thermoelectric materials. However, it is compensated by intrinsically very low values of the thermal conductivity λ, below ~0.5 W·m^−1^·K^−1^ at high temperatures [12]. Therefore, large values of the thermoelectric figure of merit ZT, defined as ZT = S^2^T/ρλ, are reached, as high as 1.2 at 650 °C in optimally doped samples [11], and 1.4 at 650 °C in optimally doped samples with the grains aligned using a texturation process [20]. These values are among the best ever observed for Pb- or Te-free p-type bulk polycrystalline materials in this temperature range. Regarding the tellurium analogue BiCuTeO, the best thermoelectric properties reported to date are not as good as the ones observed in BiCuSeO-based compounds [21], probably due to a difference in the defect chemistry leading to possible unintentional doping and to larger carriers concentrations [22], which makes the optimization of the power factor difficult. To the best of our knowledge, the thermoelectric performances of bulk BiCuSO-based samples have not been evaluated to date. We have previously studied the influence of the substitution of Se by Te in BiCuSeO [23]. Our goal was to improve the electrical properties of the material by increasing the covalent character of the Cu-*Ch* bond and tentatively decreasing the distortion of the Cu*Ch*_4_ tetrahedra. We have shown that a complete BiCuSe_1−x_Te_x_O solid solution exists between the two end-members, but we observed a strong increase of the electrical resistivity for the intermediate compositions, which degraded the thermoelectric performances [23]. However, it has been shown that an improvement of the thermoelectric figure of merit of BiCuSeO can be obtained for low substitution levels, with ZT reaching 0.7 at 650 °C in BiCuSe_0.94_Te_0.06_O, which was explained by a change in the electronic band structure [24]. In a similar manner, it would be of significant interest to investigate the influence of the substitution of selenium with sulfur on the crystal structure and transport properties of BiCuSeO-based materials, which could also lead to a decrease of the raw material price. Therefore, in this paper, we report on the synthesis, the structure, and the transport properties of the BiCuSeO-BiCuSO solid solutions.

## 2. Results and Discussion

**Figure 1:**
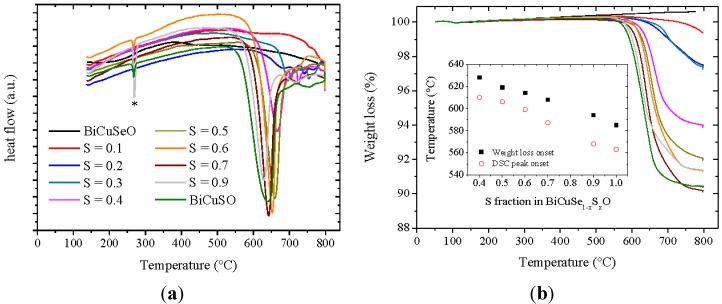
Differential scanning calorimetry curves of BiCuSe_1−x_S_x_O powders under argon (**a**) and thermogravimetric analysis of BiCuSe_1−x_S_x_O powders under argon (**b**). The peak marked with a * in the DSC curves corresponds to the melting of Bi. The inset shows the onset of the DSC peak corresponding to the decomposition of BiCuSe_1−x_S_x_O and the onset of weight loss.

As mentioned in the experimental section, the thermal treatment for the synthesis of the samples belonging to the BiCuSe_1−x_S_x_O solid solution needed to be optimized. Indeed, the decomposition temperature of the ZrCuSiAs phase depends on the actual composition of the samples, as it can be observed in Figure 1, which shows the differential scanning calorimetry and thermogravimetric analysis curves of BiCuSe_1−x_S_x_O powders under flowing argon. No DSC peak can be observed for BiCuSeO up to 790 °C, which confirms the stability of this compound up to about 800 °C. When increasing the sulfur content to x = 0.3, a clear change of the slope of the curve is present below about 700 °C, although no clear peak can be seen. Above x = 0.4, a well-resolved peak is clearly observed, and the onset temperature of this peak decreases when the sulfur content increases, down to 560 °C for BiCuSO. It shows that the stability of the phase is strongly reduced when substituting selenium by sulfur. Besides, a small peak is observed around 270 °C when the fraction of sulfur exceeds 0.4, which can be explained by the presence of a faint amount of Bi in the samples, see later. This decrease of the decomposition temperature of BiCuSe_1−x_S_x_O when increasing the sulfur fraction is correlated to volatilization in sulfur-rich samples. No weight loss is observed in the TGA curve of BiCuSeO up to 790 °C, which is consistent with our previous study reporting a good stability of this compound *versus* volatilization under argon [25]. However, this is not the case when the fraction of sulfur increases, which leads to an increase of the weight loss and to a decrease of the temperature triggering the onset of weight loss, as it can be seen in the inset of Figure 1. Therefore, as the volatilization could probably be slightly reduced during the synthesis in sealed silica tubes as compared to flowing argon, a synthesis temperature as large as 600 °C could be used, whereas the SPS temperature had to be limited to 500 °C for the sulfur-containing samples, which contrasts with BiCuSeO that can be synthesized at higher temperature [9]. Although the volatilization is unambiguously linked to the presence of sulfur, we have not been able to determine precisely the nature and amount of the elements lost. Indeed, XRD patterns recorded after the thermogravimetric analysis of BiCuSO have shown that the main residual phase is Bi, with a minor presence of poorly crystallized Cu_2-x_S and possibly a bismuth oxide and/or a mixed bismuth-selenium oxide (see Figure S1). Moreover, the total weight loss observed during the thermogravimetric analysis does not correspond to the total mass fraction of sulfur in the compounds. Therefore, it is not possible to determine precisely which element(s) is lost besides sulfur and with which proportion, which could have been used to partly compensate the volatilization during the synthesis process, although the limited amount of oxygen-containing phases after DSC (Figure S1) could be an indication of S_x_O_y_ losses. Moreover, it is not possible to determine whether the decomposition of sulfur-rich samples is due to the volatilization of some elements, or the decomposition leads to degradation-products that volatilize. Besides giving clues for the synthesis of the BiCuSe_1−x_S_x_O compounds, these results show that sulfur-substituted samples are much less stable than sulfur-free BiCuSeO, even under argon and with S fraction as low as 20%. Therefore, although the use of sulfur to substitute selenium could lead to a significant decrease of the raw material price of the thermoelectric oxychalcogenides, this lack of stability could preclude its industrial use without appropriate coatings to limit the sulfur volatilization and the materials degradation.

**Figure 2:**
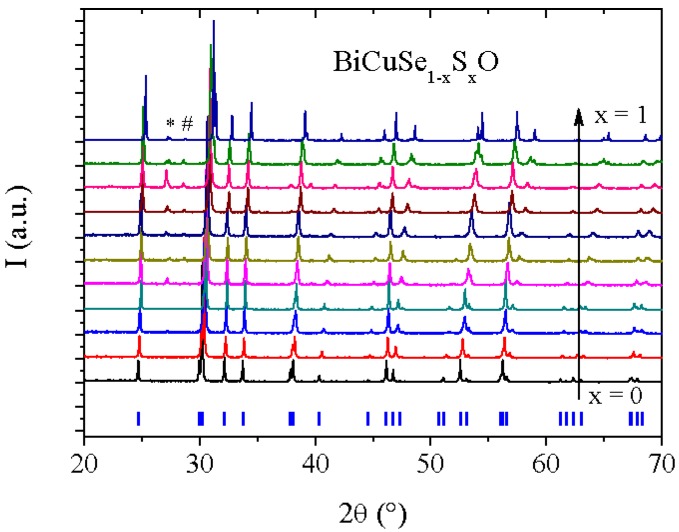
XRD patterns of BiCuSe_1−x_S_x_O samples. The ticks below the patterns indicate the Bragg positions of BiCuSeO. The peak marked with a * corresponds to Bi, and we have not been able to attribute the one marked with a #.

Figure 2 shows the XRD patterns of the samples belonging to the BiCuSe_1−x_S_x_O solid solution. Both BiCuSeO and BiCuSO crystallize in the ZrCuSiAs structure type with P4/nmm space group, and consist in [Bi_2_O_2_] and [Cu_2_Ch_2_] layers stacked along the c axis of the tetragonal unit cell. Between x = 0 and x = 0.3, the samples are well-crystallized and are single phase, within the detection limit of the XRD. When x exceeds 0.4, two supplementary peaks can be observed in the diffraction patterns, one unattributed and the other one corresponding to Bi, which is consistent with the small endothermic peak observed in the DSC curves at ~270 °C. However, all samples remain well-crystallized, and a monotonous shift of the Bragg peaks can unambiguously be observed, which shows that a complete solid solution exists between the end members BiCuSeO and BiCuSO after sufficient heating time. As the presence of the secondary phases for x > 0.4 could have originated from insufficient annealing time due to lower reaction temperature as compared to BiCuSeO, we have tried to use longer heating times or successive annealing with intermediate grinding, but no improvement of the quality of the samples was observed. Worst, the amount of secondary phases tended to increase with successive grinding and annealing, which rather suggests that the presence of these phases could be linked to the volatilization occurring in sulfur-rich samples during the thermal treatment. All attempts to obtain single phase samples for sulfur-rich compositions have been unsuccessful, with always a few percent of secondary phases in the samples (<3% of Bi except for x = 0.8 where it reaches about 8%).

**Figure 3:**
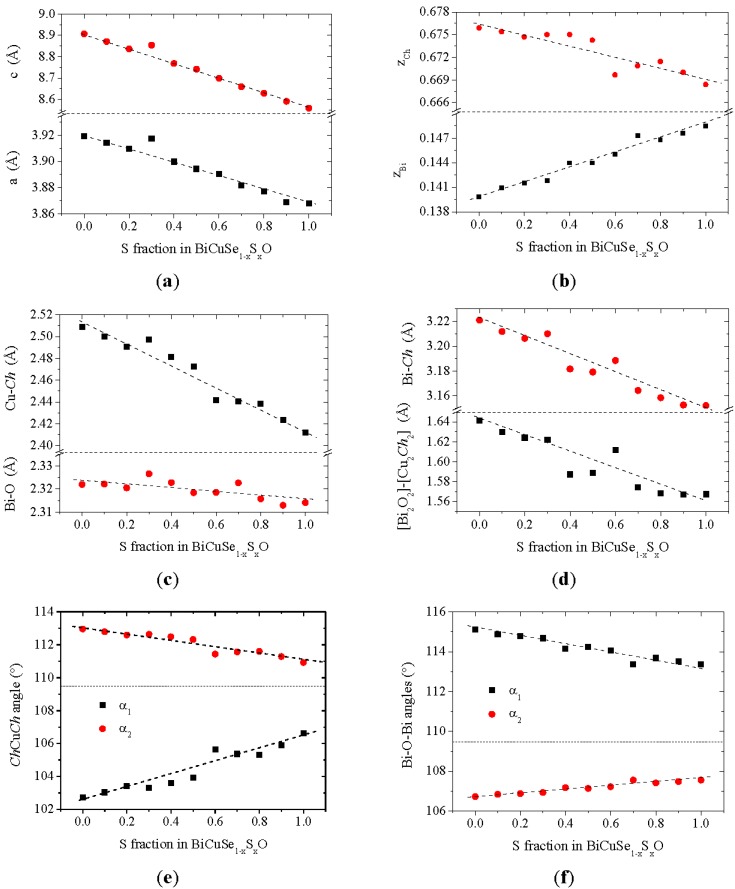
Structural parameters of the BiCuSe_1−x_S_x_O samples: lattice parameters (**a**); Bi and *Ch* atomic positions (**b**); Bi-O and Cu-*Ch* distances (**c**); Bi-*Ch* distance and distance between [Bi_2_O_2_]-[Cu_2_*Ch*_2_] planes (**d**); Cu*Ch*_4_ tetrahedra angles (**e**) and OBi_4_ tetrahedra angles (**f**).

The lattice parameters obtained using Rietveld refinement of the XRD patterns are plotted in Figure 3a. The lattice parameters of the end members of the solid solution are a = 3.919 Å, a = 3.868 Å and c = 8.907 Å, c = 8.559 Å for BiCuSeO and BiCuSO respectively. They are consistent with the values previously reported in the literature [8]. Regarding the compounds belonging to the BiCuSe_1−x_S_x_O solid solution, the dependence of their lattice parameters on the sulfur fraction can be well described using Vegard’s law. The strong decrease of both *a* and *c* is consistent with the atomic radius of Se being much larger than that of S. However, the evolution of the lattice parameters is strongly anisotropic, as the relative decrease of *c* (3.9% between BiCuSeO and BiCuSO) is much larger than the relative decrease of *a* (1.3% between BiCuSeO and BiCuSO). This anisotropic evolution of the lattice parameters in the solid solution can be related to the layered structure of the ZrCuSiAs phase. Indeed, as the substitution of selenium by sulfur is an isovalent substitution, which takes place only in the [Cu_2_*Ch*_2_] layer of the structure, it should not significantly influence the local structure of the [Bi_2_O_2_] layer, as the Bi-O ionic bond should not be significantly affected. As it can be seen in Figure 3c, the Bi-O distance is almost unchanged (the decrease of this distance is about 0.3% between BiCuSeO and BiCuSO), which limits the decrease of *a* as compared to *c*. The almost unchanged Bi-O distance is compensated by a large change in the position of the Bi atoms in the unit cell (z_Bi_ strongly increases between BiCuSeO and BiCuSO as it can be observed in Figure 3b), which is due to a slight change in the OBi_4_ tetrahedra angles whose distortion decreases (Figure 3f). The large decrease of *c* coupled with the slight evolution of the geometry of the OBi_4_ tetrahedra leads to a strong decrease of the distance between the Bi plane and the *Ch* plane and to a decrease of the Bi-*Ch* distance, by 4.5% and 2.1% respectively between BiCuSeO and BiCuSO. Although this decrease of the Bi-*Ch* distance is significant, it is rather limited as compared to the one that could have been expected by the complete substitution of selenium by sulfur, which can be roughly estimated as 6% by using the sum of Bi^3+^ ionic radius and *Ch* covalent radii (the order of magnitude of this estimation would remain the same if using other radii). These ones were chosen because previous electronic structure calculations showed that the formal charge of bismuth in BiCuSeO is large, which can be explained by the ionic nature of the Bi-O bond in the [Bi_2_O_2_] layer, whereas that of selenium is moderate, which can be explained by the covalent nature of the Cu-Se bond in the [Cu_2_Se_2_] layer [9]. This limited decrease means that the overlap between the *Ch*-np and the Bi-6p orbitals should decrease when increasing the sulfur fraction, possibly leading to an increase of the 2D characters of the electrical transport in this material. This evolution is opposite to the one reported previously for the BiCuSe_1−x_Te_x_O solid solution [23].

As the radius of sulfur is smaller than that of selenium, the Cu-*Ch* distance decreases when Se is substituted for S by 3.9% between BiCuSeO and BiCuSO (Figure 3c). This decrease of the Cu–*Ch* distance is coupled to an evolution of the geometry of the Cu*Ch*_4_ tetrahedra. Indeed, as the relative decrease of *c* is larger than the relative decrease of *a* (Figure 3a), the Cu*Ch*_4_ tetrahedra are “compressed” along the *c* axis, which leads to a significant decrease of their distortion (Figure 3e), although the chemical pressure applied when completely substituting selenium by sulfur is not sufficient to obtain regular tetrahedra. It has been previously shown using band structure calculations that the electrical properties of BiCuSeO are mostly driven by the [Cu_2_Se_2_] layer, with an almost equal contribution of the Cu-3d and Se-4p orbitals to the density of states close to the Fermi level [8,23]. Therefore, the structural evolution observed here when substituting Se with S could lead to changes in the band structure, and therefore the transport properties. On the one side, the change in the geometry of the Cu*Ch*_4_ tetrahedra should influence the overlap between the Cu-3d and Se-4p orbitals, as both d and p orbitals are directional, and the nature of the bands close to the Fermi level could be affected. On the other side, the decrease of the Cu-*Ch* distance (3.9% between the two end members of the solid solution) is smaller than the one that could be expected, which can be roughly estimated as 5.5% by using the sum of the Cu and *Ch* covalent radii. It should result in a decrease of the Cu 3d-*Ch n*p overlap, leading to a narrowing of the Cu-*Ch* antibonding levels and less dispersive bands, with a slightly lower covalent character of the Cu-*Ch* bonds.

**Figure 4:**
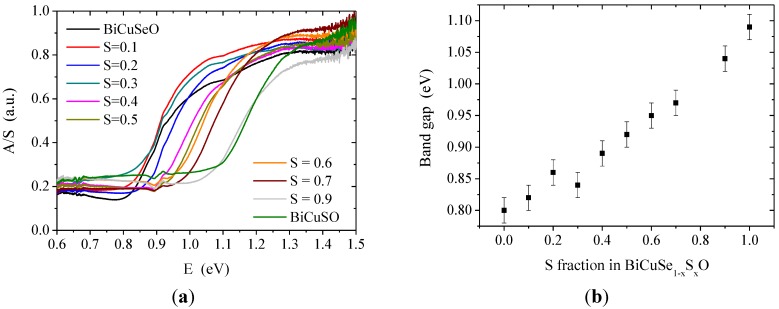
UV-Vis spectroscopy (**a**), for the definition of the *y*-axis, see text and evolution of the band-gap in the BiCuSe_1−x_S_x_O solid solution (**b**). The spectrum corresponding to BiCuSe_0.2_S_0.8_O was not recorded due to the two large amounts of secondary phases in this compound. The small feature in the spectra around 0.9 eV is due to a small glitch in the baseline, see Figure S2 in the supplementary information.

As expected, there is an evolution with the substitution of selenium with sulfur of the band gap determined by UV-Vis spectroscopy. The diffused reflectance spectra observed at room temperature where converted using Kubelka-Munk relation:

(1) $$\frac{A}{S}\;=\;\frac{{\left(1-R\right)}^{2}}{2R}$$

with R the absolute reflectance of the sample; A the molar absorption coefficient; and S the scattering coefficient. As the Kubelka-Munk relation assumes a thick infinitely diluted sample in a non-absorbing matrix with a diffuse-diffuse geometry, which was not the case here, the absolute values obtained for A/S do not have a physical meaning. However, the optical band gap can be estimated confidently through the band-edge structure. A clear band-edge structure can be observed in Figure 4, which indicates that the band gap is in the near-infrared region for all samples. The estimated band gap is 0.8 eV for BiCuSeO and 1.1 eV for BiCuSO, which is consistent with the values reported in the literature for these two compounds [8]. Within the solid solution, the band gap monotonously increases when increasing the sulfur fraction, with an almost linear trend.

The structural parameters of BiCuSeO and BiCuSO obtained using the *ab initio* calculations are summarized in Table 1. The lattice parameters agree reasonably well with the experimental data, although they are slightly larger, 2.2% and 4.3% respectively for *a* and *c* in the case of BiCuSeO, and 1.2% and 3.4% in the case of BiCuSO. However, the experimental trends, namely a decrease of the Cu-*Ch* and of the Bi-*Ch* distances slightly smaller than the ones expected from the evolution of the *Ch* radii and a decrease of the distortion of the Cu*Ch*_4_ tetrahedra, are still observed with the structural parameters obtained by the calculations. Another calculation of the electronic band structure has been performed by setting the structural parameters to the experimental values, but no significant difference was observed between the results (except slightly larger Mulliken overlap populations due to reduced distances).

The atomic valence charges resulting from a Mulliken population analysis are shown in Table 1, and the Mulliken population overlap for BiCuSeO and BiCuSO are summarized in Table 2. As already mentioned [9], the charges are far from the ones expected from a simple ionic model, namely Bi^2+^, Cu^+^, *Ch*^−^ and O^2−^, which proves a mostly covalent bonding between copper and selenium/sulfur, as compared to the Bi–O bonding, which is partly ionic, and a charge transfer between the [Bi_2_O_2_] and the [Cu_2_*Ch*_2_] layers. The covalent behavior of the Cu-*Ch* bonding is confirmed by the large calculated Cu-*Ch* Mulliken overlap population. As expected from the evolution of the Cu-*Ch* distances when substituting Se with S (see above), there is a decrease of the covalent character of the Cu-*Ch* bond when going from Se to S, which is evidenced by the decrease of the overlap population from 0.116 for Cu-Se to 0.081 for Cu-S. It results in an increase of the absolute value of the charges of Cu and *Ch*. As mentioned previously [9], the interlayer bonding in BiCuSeO is partly covalent, which is evidenced by the moderate Bi-Se overlap population. As expected from the evolution of the Bi-*Ch* distance with the substitution (see above), this overlap is strongly reduced in BiCuSO as compared to BiCuSeO, corresponding to an increase of the ionic character of the interlayer bonding.

**Table 1:** Structural parameters and atomic charges obtained by the *ab initio* calculations.

Element	x	y	z	Charge	Element	x	y	z	Charge
Bi	0.2500	0.2500	0.1316	+1.50	Bi	0.2500	0.2500	0.1423	+1.33
Cu	0.7500	0.2500	0.5000	+0.23	Cu	0.7500	0.2500	0.5000	+0.32
Se	0.2500	0.2500	0.6789	−0.63	S	0.2500	0.2500	0.6697	−0.73
O	0.7500	0.2500	0.0000	−1.10	O	0.7500	0.2500	0.0000	−0.92
Lattice parameters			a = 4.004 Å		Lattice parameters			a = 3.9149 Å	
			c = 9.2873 Å					c = 8.8536 Å	

**Table 2:** Mulliken population analysis.

Element 1	Element 2	Calc Distance (Å)	Mulliken Overlap Population	Element 1	Element 2	Calc Distance (Å)	Mulliken Overlap Population
Bi	O	2.346	0.002	Bi	O	2.328	0.004
	Se	3.333	0.036		S	3.230	0.017
Cu	Se	2.601	0.116	Cu	S	2.467	0.081
	Cu	2.831	0.045		Cu	2.768	0.031

**Figure 5:**
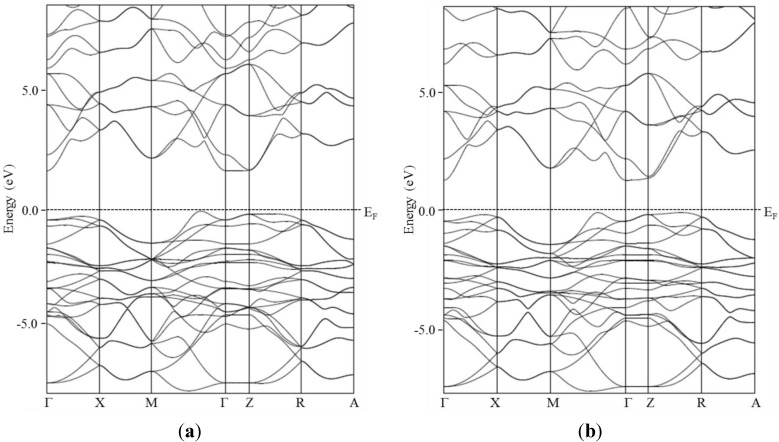
Electronic band structure close to the Fermi level of BiCuSO (**a**) and BiCuSeO (**b**).

The electronic band structures close to the Fermi level of BiCuSeO and BiCuSO are plotted in Figure 5. Both of them are consistent with the ones previously reported in the literature [8,9]. The band gap is slightly larger in BiCuSO than in BiCuSeO, which is consistent with the experimental observations. Except for the increase of the band gap when substituting Se with S, both electronic band structures are almost the same, which was somehow unexpected because of the evolution of the structural parameters (decrease of the distortion of the CuCh4 tetrahedra and evolution of the distances between Cu and *Ch* and Bi and *Ch*). Therefore, electrical transport properties should not be affected very much by the substitution.

**Figure 6:**
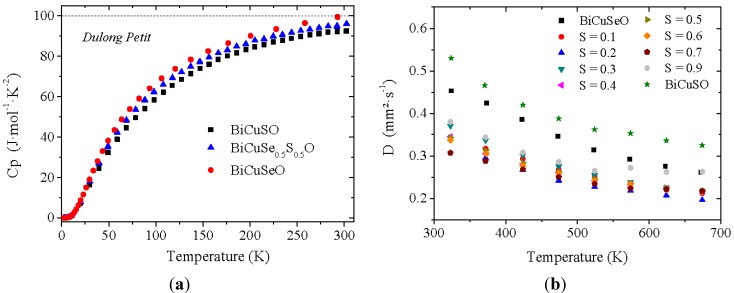
Temperature dependence of the specific heat (**a**), for the clarity of the figure, only 3 curves were plotted, and temperature dependence of the thermal diffusivity of BiCuSe_1−x_S_x_O pellets (**b**).

Figure 6a shows the specific heat of the samples belonging to the BiCuSe_1−x_S_x_O solid solution (for the clarity of the figure, only 3 curves were plotted). As expected, for unintentionally doped semiconductors, the Sommerfeld coefficient γ, which is linked to the density of states at the Fermi level by the relation:

(2) $$\text{γ}=\frac{{\text{π}}^{2}{k_{B}}^{2}}{3}D(E_{F})(1+{\text{λ}}_{e-ph})$$

where λ_e-ph_ is the electron-phonon coupling constant, is very close to zero. A slight difference can be noticed between the different samples: the specific heat of BiCuSeO reaches the Dulong-Petit limit close to room temperature, whereas it is not the case for BiCuSO. Moreover, the specific heat of BiCuSO is slightly lower than that of BiCuSeO in the whole temperature range, and the intermediate compound BiCuSe_0.5_S_0.5_O lies in between. More generally, the room temperature value of C_p_ decreases when increasing the sulfur fraction. This result indicates an increase of the Debye temperature θ_D_ when substituting Se for S, which is consistent with the values calculated in the literature for BiCuSeO (243 K) [12] and BiCuSO (289 K) [26].

**Figure 7:**
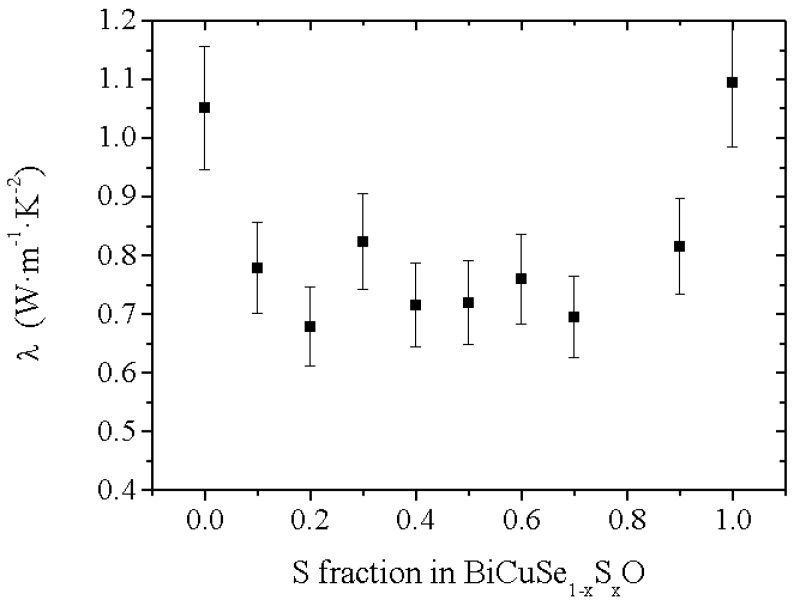
Evolution of the room temperature value of the thermal conductivity in the BiCuSe_1−x_S_x_O solid solution.

The thermal conductivity in the BiCuSe_1−x_S_x_O solid solution has been calculated close to room temperature using the interpolated values of the measured specific heat, thermal diffusivity (Figure 6b) and geometrical density, and the values are plotted in Figure 7. As the electrical resistivity at room temperature is large (>200 mΩ.cm in the most conductive sample), see later, the thermal conductivity plotted here is almost only constituted by the lattice contribution λ_lat_. The thermal conductivity of BiCuSeO is consistent with the value already reported for this compound, with λ~1 W·m^−1^·K^−1^ [10]. Interestingly, the room temperature thermal conductivity value of BiCuSO is almost the same within the uncertainty of the measurement, despite a significantly larger thermal diffusivity, because of both lower specific heat and theoretical density. However, the thermal conductivity is strongly reduced for the intermediate compositions. As the microstructure of all samples is micron-size, with no influence of the composition on the grain size or the preferential orientation, the lower values for the intermediate compositions as compared to the end members could not be explained solely by a change in grain-boundary scattering of the phonons. Moreover, as no impurity phase can be detected between S = 0 and S = 0.3, the decrease of λ from BiCuSeO to BiCuSe_0.7_S_0.3_O cannot originate from impurity scattering of the phonons. However, this decrease can be explained by the diffusion of the phonons by point defect scattering [27] due to the mass and volume difference between Se and S, and it shows that the partial substitution of Se by S to form the BiCuSe_1−x_S_x_O solid solution is a good strategy to decrease the thermal conductivity of the thermoelectric oxychalcogenides.

**Figure 8:**
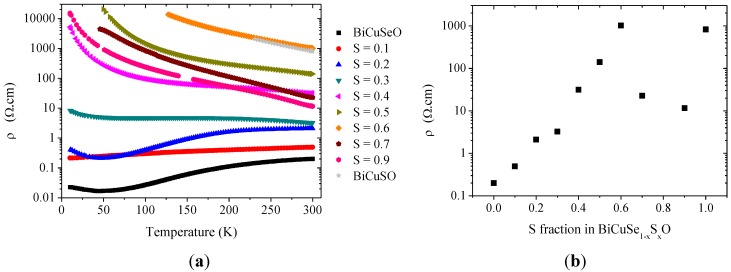
Temperature dependence (**a**) and evolution of the room temperature value of the electrical resistivity (**b**) in the BiCuSe_1−x_S_x_O solid solution.

As the electronic band structures of BiCuSeO and BiCuSO are very similar, with the most significant difference being the slight increase of the band gap, no major evolution of the electrical resistivity was expected within the solid solution. However, it is clearly not the case, as it can be observed in Figure 8, which shows the temperature dependence and the room temperature values of the electrical resistivity in the solid solution, and as it had already been reported by Hiramatsu *et al.* [8]. The electrical resistivity of BiCuSeO exhibits a “bad metal” behavior, with room temperature ρ_300K_~200 mΩ.cm and a RRR = 12, as already reported [8,9]. It has been shown that this behavior, rather unexpected for a semiconductor with a band-gap of 0.8 eV, can be well explained by an unintentional doping due to the presence of faint amount of copper vacancies [8,9], which has been confirmed both experimentally [19] and theoretically [28], although it could also be linked to a more complex defect chemistry with complex Cu and Se defects [29]. When Se is partly substituted by S, the rough tendency is an increase of the electrical resistivity coupled to a change to a semiconductor behavior, consistent with the band-gap of 1.1 eV observed in BiCuSO (the relatively large dispersion of the data for S > 0.6 originates from the presence of Bi secondary phase with low resistivity in the samples for large sulfur fractions, most especially for x = 0.7 and x = 0.9, as it has been observed in the XRD patterns). The increase of the room temperature value is very large, about 4 order of magnitude between x = 0 and x = 0.6. We have tried to evaluate the evolution of the holes concentration in the solid solution using Hall effect measurements, but the samples revealed themselves to be too resistive for an accurate determination of the Hall coefficient. However, we can reasonably hypothesize that the large increase of the electrical resistivity when the sulfur fraction increases is caused by a large decrease of the holes’ concentrations in the samples. This decrease could originate from a decrease of the copper vacancies’ concentrations with sulfur substitution, due to an evolution of the energy of formation of this acceptor defect. Indeed, a large evolution of the energy of formation of Cu and Bi vacancies has been recently suggested in Bi_1−x_Pb_x_CuTeO by DFT calculations, for Pb concentrations as low as 0.1 [22]. Therefore, a similar evolution could be observed in BiCuSe_1−x_S_x_O.

As the electrical resistivity strongly increases with the substitution of Se by S, the thermoelectric performances of the materials are obviously largely degraded. However, it should be reminded that this substitution is isovalent and that all samples are far from the optimum carriers concentration, which is close to 10^21^ cm^−3^ in BiCuSeO [9,11]. Therefore, the electrical properties would obviously be improved by aliovalent doping, for example using a 2+ cation to substitute Bi. Coupled to ~25% reduction of the thermal conductivity in intermediate compositions of the BiCuSe_1−x_S_x_O solid solution, it could lead to an increase of the thermoelectric figures of merit ZT as compared to sulfur-free compounds.

## 3. Experimental Section

BiCuSe_1−x_S_x_O (x = 0–1) samples were synthesized by a two-step solid-state reaction route. A stoichiometric mixture of Bi_2_O_3_ (4 N), Bi_2_S_3_ (3 N), Bi (3 N), Cu (3 N), Cu_2_S (3 N) and Se (5 N) powders was mixed in a mortar and pressed into bars, which were then sealed in silica tubes, under vacuum, and heated at 350 °C for 15 h. Many different thermal treatments were attempted in order to obtain good quality samples. In the optimized one, the obtained materials were crushed into powders, and then cold pressed into bars and heated again at 600 °C for another 1 week in sealed silica tubes. After that, obtained bars were ground into powders, the obtained powders were densified by a spark plasma sintering system (Sumitomo SPS1050; Sumitomo Coal Mining Company, Ltd., Tokyo, Japan) under an axial compressive stress of 100 MPa in Ar at 500 °C for 10 min, resulting in a disk-shaped sample of Ø 15 mm × 4 mm.

Room temperature X-ray diffraction characterization was performed using a Panalytical X’Pert diffractometer by using a Cu–K_α1_ radiation, with a Ge(111) incident monochromator and a X’celerator detector. Rietveld refinement was performed using FULLPROF software [30].

Room temperature optical diffuse reflectance measurements were performed on finely ground powders. The spectra were collected in a Varian Cary 5000 double-beam, double-monochromator spectrophotometer (Ultraviolet-Visible diffuse reflectance Spectra), with a PTFE integrating sphere.

Thermogravimetric analysis (TGA) and differential scanning calorimetry (DSC) were performed using a Setaram Setsys Evo under an Ar atmosphere in the temperature range from room temperature to 1073 K with a heating rate of 5 K·min^−1^.

The specific heat C_p_ was measured using a Quantum Design PPMS (physical properties measurement system) from 300 K to 2 K using apiezon N grease. The typical size of the samples was 0.2 × 0.2 × 0.1 mm^3^, which corresponds to about 30 mg. The thermal diffusivity (D) was measured up to 673 K in the thickness direction of a square sample of 8 × 8 mm^2^ and 1–2 mm in thickness by using a laser flash diffusivity method (LFA457; NETZSCH, Selb, Germany). The room temperature thermal conductivity (λ) was calculated by the relation λ = DC_p_d; where C_p_ is the specific heat and d is the density (calculated using the dimension and weight of the pellets). The electrical resistivity ρ was measured on bars cut from the pellets, with a typical size of 10 × 2 × 2 mm^3^, with a standard 4-probes method in a closed-cycle cryostat.

All the calculations were performed using CRYSTAL09, an ab initio code for periodic systems, developed in Turin [31]. The crystalline orbitals are expanded in terms of localized atomic Gaussian basis set, in a way close to the linear combination of atomic orbitals (LCAO) method currently adopted for molecules. The eigenvalues equations are solved at the B3LYP level. The hybrid B3LYP functional uses the Becke’s exchange [32] and Lee–Yang–Parr’s correlation functional [33]. The number of k points in the first irreducible Brillouin zone at which the hamiltonian matrix is diagonalized was equal to 75. In order to reduce the computational cost, the Hay and Wadt large core [34] and the Durand and Barthelat effective core pseudopotentials [35] were used to model the core electrons in selenium/copper and bismuth, respectively. Valence basis sets already optimized in early studies were adopted 8-411d11G for oxygen [36]. CRYSTAL09 code carries out the optimization of any parameter (geometrical parameters or atomic positions) relative to the total energy of the system by a conjugated gradient algorithm.

## 4. Conclusions

In this study, we have shown that a complete solid solution exists between BiCuSeO and BiCuSO. The increase of the sulfur fraction in the materials led to a strong decrease of the stability, linked to volatilization. Therefore, the use of S-substituted BiCuSeO-based materials for thermoelectric applications would require the development of a protective coating to prevent their degradation. With the substitution of selenium by sulfur, the distortion of the Cu*Ch*_4_ tetrahedra decreased. Meanwhile, the covalent character of the Cu-*Ch* bond decreased, as well as the overlap between Bi 6p and *Ch n*p orbitals, which lead to an increase of the ionic character of both intralayer and interlayer bonding in this layered material. Although the thermal conductivity is nearly the same in BiCuSeO and BiCuSO, it is significantly reduced for intermediate compositions, by about 25% due to point defect scattering. However, a strong increase of the electrical resistivity has been observed when increasing the sulfur fraction, which may be linked to a change in the energy of formation of copper vacancies. As the optimum carriers concentration in BiCuSeO is of the order of 10^21^ cm^−3^, the optimization of the thermoelectric properties would require the doping of the materials. If a similar power factor could be obtained in S-substituted samples to the one observed in BiCuSeO, the strong decrease in the thermal conductivity would lead to improved thermoelectric performances.

## Supplementary Materials

Supplementary materials can be accessed at: http://www.mdpi.com/1996-1944/8/3/1043/s1.
