# Supplementary material for: Structure and Transport Properties of the BiCuSeO-BiCuSO Solid Solution
Source: Materials (Basel). 2015 Mar 12;8(3):1043–58. doi: 10.3390/ma8031043 (PMC5455453; doi:10.3390/ma8031043)
Supplement: Supplementary file 1 [file materials-08-01043-s001.pdf]

Supplementary Materials

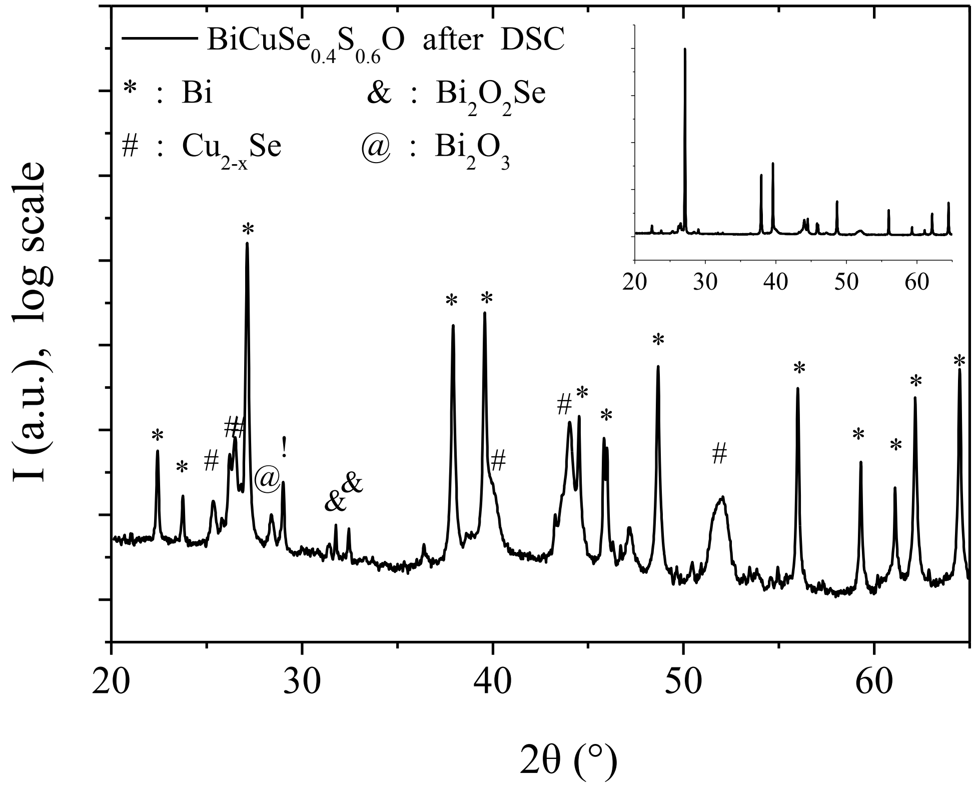

Figure S1. XRD pattern of $BiCuSe_{0.4}S_{0.6}O$ after the DSC treatment, with a tentative assignment of the peaks. Inset: same XRD pattern in linear scale.

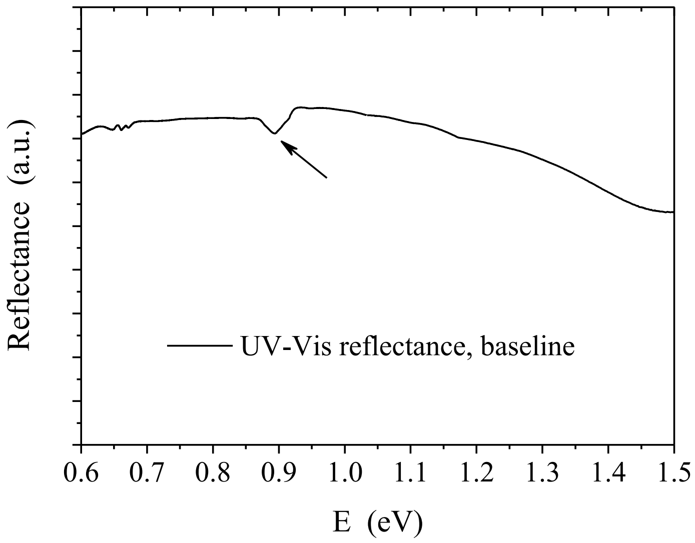

Figure S2. Baseline of the UV-Vis reflectance spectra.
